# Protocol for a quasi-experimental, 950 county study examining implementation outcomes and mechanisms of Stepping Up, a national policy effort to improve mental health and substance use services for justice-involved individuals

**DOI:** 10.1186/s13012-021-01095-2

**Published:** 2021-03-29

**Authors:** Jennifer E. Johnson, Jill Viglione, Niloofar Ramezani, Alison E. Cuellar, Maji Hailemariam, Rochelle Rosen, Alex Breno, Faye S. Taxman

**Affiliations:** 1 Division of Public Health, Michigan State University, 200 East 1st St Room 366, Flint, MI 48502 USA; 2 Department of Criminal Justice, University of Central Florida, 12805 Pegasus Drive, Orlando, FL 32816 USA; 3 Department of Statistics, George Mason University, 4400 University Drive, MS 4A7, Fairfax, VA 22030 USA; 4 Department of Health Administration and Policy, George Mason University, Fairfax, VA 22030 USA; 5 Department of Obstetrics, Gynecology, and Reproductive Biology, Michigan State University, 965 Wilson Road, A631B, East Lansing, MI 48824 USA; 6 Center for Behavioral and Preventive Medicine, The Miriam Hospital, Providence, RI USA; 7 Center for Advancing Correctional Excellence, George Mason University, 4400 University Drive, Fairfax, VA 22030 USA; 8 Schar School of Policy & Government, Center for Advancing Correctional Excellence, George Mason University, 4400 University Drive, Fairfax, VA 22030 USA

**Keywords:** Implementation, Jail, Mental health, Substance use, Outcome, Mechanism, Community

## Abstract

**Background:**

The criminal justice system is the largest provider of mental health services in the USA. Many jurisdictions are interested in reducing the use of the justice system for mental health problems. The national Stepping Up Initiative helps agencies within counties work together more effectively to reduce the number of individuals with mental illness in jails and to improve access to mental health services in the community. This study will compare Stepping Up counties to matched comparison counties over time to (1) examine the effectiveness of Stepping Up and (2) test hypothesized implementation mechanisms to inform multi-agency implementation efforts more broadly.

**Methods:**

The study will survey 950 counties at baseline, 18 months, and 36 months in a quasi-experimental design comparing implementation mechanisms and outcomes between 475 Stepping Up counties and 475 matched comparison counties. Surveys will be sent to up to four respondents per county including administrators of jail, probation, community mental health services, and community substance use treatment services (3800 total respondents). We will examine whether Stepping Up counties show faster improvements in implementation outcomes (number of justice-involved clients receiving behavioral health services, number of behavioral health evidence-based practices and policies [EBPPs] available to justice-involved individuals, and resources for behavioral health EBPP for justice-involved individuals) than do matched comparison counties. We will also evaluate whether engagement of hypothesized mechanisms explains differences in implementation outcomes. Implementation target mechanisms include (1) use of and capacity for performance monitoring, (2) use and functioning of interagency teams, (3) common goals and mission across agencies, and (4) system integration (i.e., building an integrated system of care rather than adding one program or training). Finally, we will characterize implementation processes and critical incidents using survey responses and qualitative interviews.

**Discussion:**

There are few rigorous, prospective studies examining implementation mechanisms and their relationship with behavioral health implementation outcomes in justice and associated community behavioral health settings. There is also limited understanding of implementation mechanisms that occur across systems with multiple goals. This study will describe implementation outcomes of Stepping Up and will elucidate target mechanisms that are effective in multi-goal, multi-agency systems.

**Supplementary Information:**

The online version contains supplementary material available at 10.1186/s13012-021-01095-2.

Contributions to the literature

- This paper describes the methods for the first prospective national study evaluating implementation outcomes of efforts to help justice and community behavioral health systems work together better to keep individuals with mental illness out of jail.
- This protocol paper provides an example of how to study outcomes and mechanisms of policy implementation.
- This protocol paper provides an example of a strong quasi-experimental design that allows for testing both implementation outcomes and mechanisms when randomization is not possible, which is often the case with policy implementation.
- The quasi-experimental design is especially strong and the sample large (950 counties) for a policy implementation research study, a still understudied area of implementation science.

## Background

More than 10 million adults are arrested and enter the United States criminal justice (CJ) system, including pretrial detention, jail, probation, and parole, each year [1]. Rates of current mental health (56%) and substance use (66%) disorders are elevated among justice-involved populations [2], who disproportionately lack education, experience victimization and homelessness, and have poor employment skills, complicating care and increasing morbidity and mortality [3–13]. The CJ system is a low-resource setting charged with serving people who face complex behavioral and physical health issues, with inadequate access to health care.

Counties (which often operate jails, probation and/or parole services, community mental health, and substance use treatment systems) care for the vast majority of justice-involved individuals. Recognizing that counties are overwhelmed by overuse of incarceration for those with mental health and substance use disorders and by the lack of health services for justice-involved individuals in the community, the National Association of Counties (NACo), the Council of State Governments (CSG) Justice Policy Center, and the American Psychiatric Association Foundation (APAF) developed the Stepping Up Initiative [14]. The goal of Stepping Up is to reduce the number of individuals with serious mental illness in jails and to improve access to community mental health services for currently or potentially justice-involved individuals. To join Stepping Up [14], counties pass a resolution to reduce unnecessary use of jail and increase access to community behavioral health services using a broad, locally adaptable six-step action plan: (1) convene a diverse team of leaders, (2) collect and review data on individuals in the justice system, (3) examine treatment and service capacity, (4) develop a plan with measurable outcomes, (5) implement the plan, and (6) track ongoing progress with data. Since it began in 2015, Stepping Up has registered more than 500 counties.

This study will compare implementation outcomes and mechanisms in the first 475 Stepping Up counties to those in 475 matched comparison counties to: (1) examine the effectiveness of Stepping Up and (2) test hypothesized implementation mechanisms to inform multi-agency implementation efforts more broadly. The study team did not design Stepping Up. However, the national Stepping Up effort provides a unique opportunity for a strong quasi-experimental study examining multi-agency implementation outcomes and mechanisms hypothesized to lead to these outcomes.

## Definitions

This study uses a broad definition of mental health and substance use evidence-based practices and policies (EBPPs) including (1) behavioral and pharmacological treatments; (2) procedures such as assessment and care coordination; (3) practices such as diversion, supported employment, and problem-solving courts; and (4) policies including laws or regulations, judicial decrees, and agency guidelines. Our definition of “justice-involved individuals” is taken from the Sequential Intercept Model [15], and includes the full range of relevant county and local justice involvement (i.e., 911 calls, local law enforcement contact, pretrial jail detention, court appearances, specialty courts, jail sentences, probation, and parole). Implementation outcomes include number of justice-involved clients receiving behavioral health services, number of behavioral health EBPP available to justice-involved individuals, and resources for behavioral health EBPP for justice-involved individuals.

## Implementation mechanisms

**Fig. 1:**
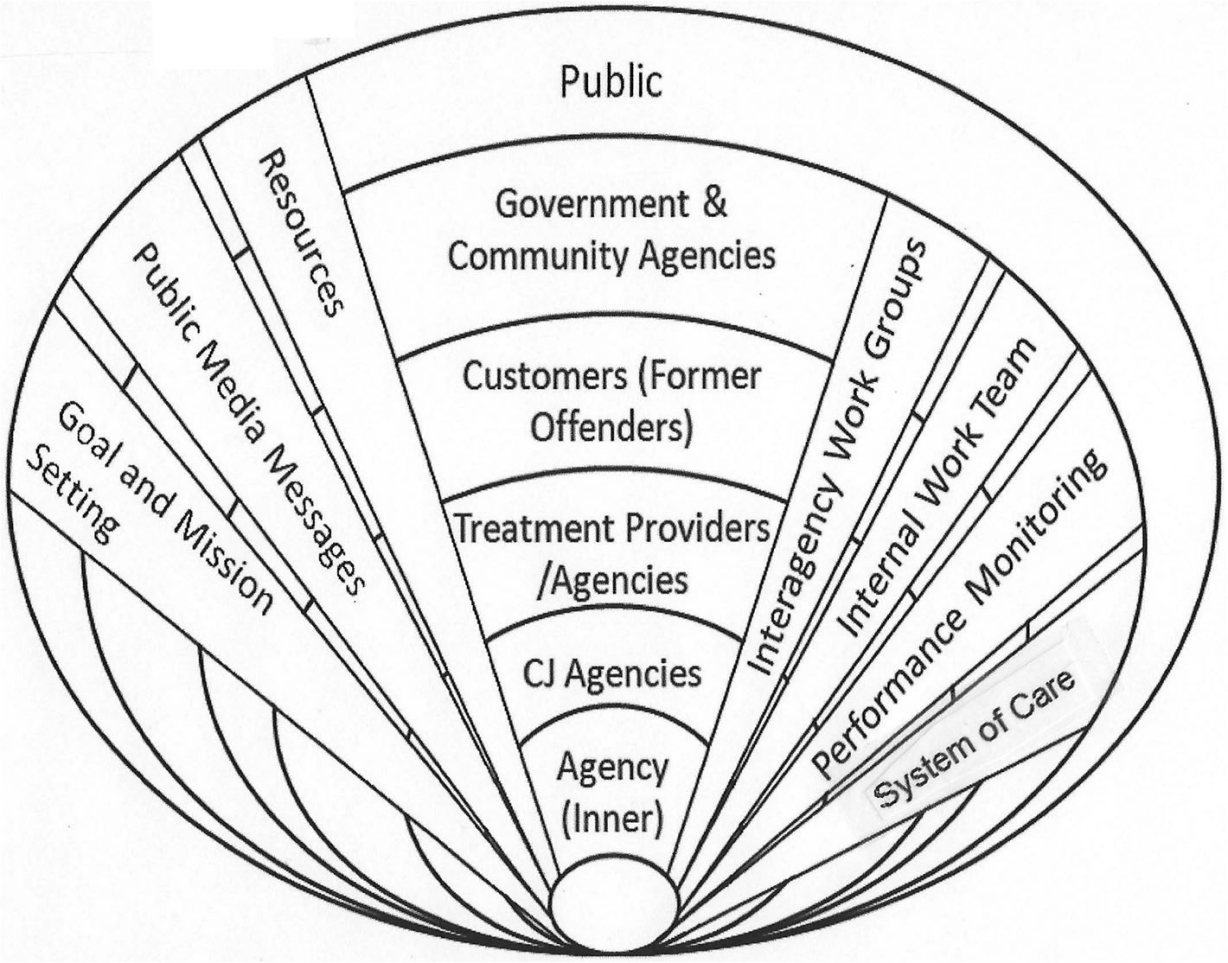
Study implementation mechanisms are taken from the Criminal Justice Interagency Implementation Model (IIM). Figure adapted from Taxman et al. [17]

Recent work has emphasized the importance of identifying implementation mechanisms underlying observed implementation outcomes [16]. Identifying implementation mechanisms can inform future development of more powerful implementation interventions. Lewis et al. define implementation mechanisms as “processes or events through which an implementation strategy operates to effect desired implementation outcomes” (p.3) [16]. Mechanisms examined in this study come from the Criminal Justice Interagency Implementation Model (CJ-IIM; Fig. 1) [17], which describes the need for cooperation of multiple constituencies for CJ to pursue public health goals (such as improving mental health and substance use services). Implementation mechanisms described in the CJ-IIM address the cross-contextual-layer cooperation needed for behavioral health implementation in CJ systems. This study proposes to examine four of the CJ-IIM mechanisms (performance monitoring, interagency work groups, goal and mission setting across agencies, and system integration) as mechanisms of action for Stepping Up (Table 1).

**Table 1:** Study constructs, function, relation to Stepping Up, and relation to the study conceptual model (CJ-IIM)

Construct	Study function	Relation to Stepping Up	CJ-IIM concept
**Target implementation mechanisms**			
Use of/capacity for performance monitoring	Mechanism	Steps 2, 3, and 6: Collect, measure and track capacity	Performance monitoring
Use and functioning of interagency teams	Mechanism	Step 1: Convene a diverse team of leaders	Interagency workgroups
Common goals and mission across agencies	Mechanism	Steps 4 and 5: Develop and implement a plan together	Goal, mission setting
System integration (vs. single programs)	Mechanism	Steps 4 and 5: Develop and implement a plan together	System of care
**Implementation outcomes**			
Number of justice-involved clients who received behavioral health services	Primary	Outcomes are indicators of county capacity to identify and treat justice-involved individuals with behavioral health problems and link them to or provide appropriate community care.	
Number of behavioral health EBPP available	Secondary		
Resources for behavioral health EBPP	Secondary		

### Use of and capacity for performance monitoring

Performance monitoring refers to the use of data to assess population needs, understand how systems work, identify desired outcomes, and monitor progress. Stepping Up recommends that counties develop capacity for ongoing monitoring of four measures: (1) number of people with mental illnesses who are booked into jail, (2) average length of stay in jail, (3) percent linked to community-based treatment after release from jail; and (4) return to jail rate (i.e., recidivism). Doing so often requires infrastructure development since justice and behavioral health agencies lack common identifiers to track clients across systems, and few jurisdictions have electronic health record systems. This study will assess whether Stepping Up efforts improve counties’ ability to track and use performance monitoring metrics (mechanism) relative to comparison counties, and whether doing so improves implementation outcomes.

### Use and functioning of interagency teams (i.e., mental health, substance use, jail, probation, county government)

Moving away from a focus on one agency to the larger system requires interagency teams to exist and to work together. The failure of agencies to function as a team limits their ability to address larger, system-wide issues. The Stepping Up initiative explicitly promotes an interagency stakeholder model. The first step prescribed by the initiative is for the county to convene a team of jail, probation, community mental health, substance use treatment, and elected representatives to work together on the remaining steps. This study will assess whether Stepping Up efforts improve the use and functioning of interagency teams (mechanism) relative to comparison counties and whether use and functioning of interagency teams improve implementation outcomes.

### Having common goals and mission across agencies

EBPPs are more likely to be implemented in systems with clear, visible goals [18, 19], where the EBPPs are consistent with the agency’s mission [17, 20]. CJ agencies such as jails, police, and prosecutors have primary public safety goals, with some secondary public health responsibilities. For behavioral health EBPP implementation, cooperating systems need to incorporate behavioral health EBPPs as a vital ingredient for achieving the primary mission of public safety or agree that public safety can be better achieved through improved behavioral health [17]. For the systems to work together to address common goals, external stakeholders must support this mission shift. To join Stepping Up, counties pass a resolution providing strong, visible support for health and justice agencies to deviate from their siloed missions to create integrated goals. This study will assess whether Stepping Up increases use of cross-agency goals (mechanism) relative to comparison counties, and whether doing so explains any observed differences in implementation outcomes.

### System integration

The CJ-IIM hypothesizes that implementation efforts will be most effective as they broaden engagement and ownership across agencies to develop a county-wide system of care, rather than adding single programs or trainings. Many agencies are involved in CJ behavioral healthcare (e.g., community mental health centers, substance use treatment agencies, police, courts, jail, probation, prison, and parole). Service linkage among them is often inadequate or non-existent [21, 22]. Previous analyses of CJ reform efforts suggest that the tendency is to implement a new program rather than working toward a cohesive system of care [23, 24]. The study will examine whether Stepping Up counties are more successful at creating integrated systems of care (mechanism) than are comparison counties, and whether doing so explains any observed differences in implementation outcomes.

## Implementation mechanisms of Stepping Up (I.M. Stepping Up) study aims

**Fig. 2:**
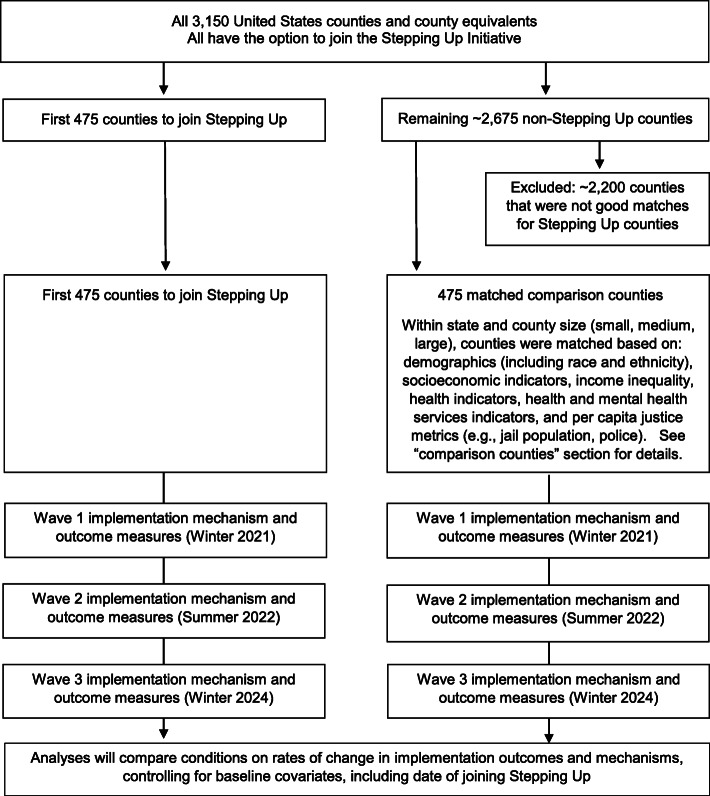
Study flow diagram

This study will compare Stepping Up counties to matched comparison counties on implementation outcomes and mechanisms (see Table 1). We will use surveys and qualitative interviews to compare 475 Stepping Up and 475 paired target counties at 3 waves: study baseline, 18 months, and 36 months (see Fig. 2). County- and agency-level data will be collected from individuals in 4 specific criminal justice and behavioral health administrator roles in each county (up to 3800 total respondents at each wave). Stepping Up and comparison counties will be compared on rates of change in engagement of target mechanisms (primary) and rates of change in implementation outcomes (secondary). Specific aims are to

1. *Compare engagement of target implementation mechanisms between Stepping Up and comparison counties.* We will examine whether (a) Stepping Up counties show a faster rate of improvement in hypothesized target mechanisms (use of/capacity for performance monitoring, use and functioning of interagency teams, common goals across agencies, and system integration) than do comparison counties; and (b) whether target mechanisms mediate effects of Stepping Up on implementation outcomes.
2. *Compare implementation outcomes between Stepping Up and comparison counties.* We will examine whether Stepping Up counties show faster rate of improvement in implementation outcomes (number of justice-involved clients receiving behavioral health services, number of behavioral health EBPP available to justice-involved individuals, and resources for behavioral health EBPP for justice-involved individuals) than do comparison counties.
3. *Characterize implementation processes and critical incidents* occurring in Stepping Up and comparison counties. We will (a) use qualitative data to triangulate quantitative findings and enrich an understanding of how the target mechanisms produce outcomes, (b) assess counties’ fidelity to Stepping Up, and (c) explore which naturally occurring implementation strategies used in 950 counties lead to better implementation outcomes.

## Innovation

Reform rhetoric is common, but there are few rigorous, prospective studies examining implementation mechanisms and their relationship with behavioral health implementation outcomes in CJ settings. The national Stepping Up Initiative, which seeks to change the interface between behavioral health services and the justice system provides a large natural experiment and a unique research opportunity.

The proposed project also has novel implications for implementation science. Most implementation change process studies have been conducted within a single organization (e.g., health). There is limited understanding of implementation mechanisms that occur within or across systems with multiple goals, where some goals may be secondary or foreign [17]. This study will inform other implementation efforts by identifying the potential target mechanisms in complex, multi-agency systems [16].

## Methods

County matching and baseline covariate controls increase the rigor for this longitudinal natural experiment, which is described consistent with STROBE guidelines [25] (see Supplemental Materials). The study will measure target mechanisms and key outcomes in 475 Stepping Up counties and 475 matched counties at three waves: study baseline, 18 months, and 36 months. Since counties self-select to be in Stepping Up, we cannot randomize counties to the Stepping Up Initiative. Instead, we use a case-matched design using the Centers for Disease Control and Prevention (CDC) peer counties methodology to identify non-Stepping Up comparison counties with similar size, demographics, and health, economic, and justice indicators [26–28]. Stepping Up began in 2015 and has been registering counties over time. Therefore, at study baseline, the 475 Stepping Up counties had been participating in Stepping Up from 1 to 5 years. Wave 1 (i.e., baseline) values and months since each county began Stepping Up will serve as covariates. Therefore, analyses examine differences between groups in within-county rates of change between study waves controlling for Wave 1. Our quantitative survey results will be augmented with qualitative interviews with 60 counties at each wave to enrich our understanding of how the implementation mechanisms work.

### The Stepping Up Initiative

The goal of Stepping Up is to reduce the number of individuals with mental illness in jails and to improve access to community mental health services for currently or potentially justice-involved individuals. To join Stepping Up [14], counties pass a resolution to address behavioral health disorders (i.e., reduce unnecessary use of jail, increase access to behavioral health services) using a broad, locally adaptable six-step action plan: (1) convene a diverse team of leaders, (2) collect and review data on individuals in the justice system, (3) examine treatment and service capacity, (4) develop a plan with measurable outcomes, (5) implement the plan, and (6) track ongoing progress with data. Table 1 illustrates how we mapped Stepping Up’s six steps onto hypothesized CJ-IIM mechanisms. Stepping Up is a manualized approach, and the national initiative has offered a variety of written and other technical assistance resources.

Stepping Up outlines steps to help counties become more data-driven. Leaders of agencies within each county are asked to agree upon a mutual definition of terms such as “mental illness”, “connection to community-based care”, and “recidivism”. Stepping Up then encourages counties to identify and use a universal, validated mental health screening instrument for new intakes into the jail and other agencies. The screening tool identifies individuals in need of a full clinical assessment. Stepping Up offers toolkits to help counties examine how to capture screening and assessment results electronically and engage in information-sharing agreements. Counties are encouraged to track data on four key measures to assess impact of their efforts over time: (1) number of people with mental illnesses who are booked into jail, (2) average length of stay, (3) percentage of people connected to community-based treatment after release from jail, and (4) rate of return to jail. With this data infrastructure in place, counties can assess the effects of their efforts to address patient needs (e.g., substance use, mental illness, family discord), continuity of care, and reach of services. As Stepping Up counties work to iteratively improve services, they can track progress, focus county leaders on key outcome measures, and make the budgetary and programmatic case for needed resources. This study will assess counties’ progress on these steps.

### Survey methods

#### Stepping Up counties

When this project was submitted for funding, there were 475 counties designated as Stepping Up counties. Although new counties continue to join Stepping Up, for feasibility, this study will assess the 475 counties originally proposed.

#### Comparison counties

A peer group of 475 matched comparison counties was created using a county grouping methodology developed by CDC [26–28] and updated by our team [29]. Matching variables were drawn from three primary data sources: (1) Vera Institute’s incarceration trends database [30] for county pretrial and jail populations; (2) County Health Rankings & Roadmaps [31] for health, economic, social, and demographic information; and (3) the Uniform Crime Report [32, 33] for crime and police data.

Counties were nested within states and clustered on health and social indicators. Therefore, a hierarchical matching approach needed to be developed to accommodate state- and county-level covariates. The study principal investigators initially chose 34 of the most potentially relevant variables from the datasets based on expert knowledge. These variables included demographic factors (e.g., median household income, unemployment, total population, high school graduation rate, percent African American, percent Hispanic), inequality indicators (e.g., income inequality, residential segregation), health factors (e.g., poor mental health days, poor physical health days, HIV incidence), healthcare (e.g., mental health providers per capita, primary care physicians per capita, percent of drug treatment paid by Medicaid), crime, and criminal justice (e.g., per capita numbers of police officers, jail population, jail pretrial population, juvenile criminal cases). Based on random forest models and team feedback, these variables were reduced to 29 total variables: 22 predictors and 7 variables reflecting jail populations and mental health providers in the area (factors central to Stepping Up activities). Third, shrinkage-based variable selection techniques were applied to select variables that best predicted jail population per capita, pretrial population per capita, and per capita rate of mental health providers, without collinearity. Next, logistic models (which included both predictors and dependent variables in the previous models) were fitted to define variable weights and estimate the likelihood of each county classifying as a Stepping Up or non-Stepping Up county. Using these weights, matching scores were calculated for each county and used in an algorithm to find the best control matched county for each Stepping Up county among potential comparison counties within the same state.

The final variables used for county-matching scores included per capita rates of mental health providers, daily jail population, daily jail pretrial population, primary care providers, police, licensed psychologists, and community mental health centers. Final variables also included average number of physically unhealthy days (of 30), high school graduation, and income inequality rates, total county healthcare expenses, percent African American population, percent Hispanic population, percent drug treatment paid by Medicaid, county population, and an indicator reflecting presence of a medical school in the county. In states where the number of Stepping Up counties was higher than the number of potential comparison counties, state location, Medicaid expansion status, and justice/mental health policy were used to pair comparable states and then algorithmically match at the county level. If a county from the comparison group joins Stepping Up during the first year of the study, we find a new matching county. If this occurs after the first year of the study, the pair will be removed from analyses.

#### Survey respondents

The overall sample is 475 Stepping Up counties and 475 comparison counties. In each county, we will survey the administrators of community mental health, jail, probation, and community substance use treatment agencies (i.e., up to 4 respondents per county and ~ 3800 total; see Table 2). These respondents were selected because the jail and probation systems have the majority of individuals under justice control in a county, and mental health and substance use treatment administrators are responsible for the provision of behavioral health services for justice-involved individuals in the community.

**Table 2:** Targeted type and number of survey respondents

Respondent type	Estimated number
Community mental health administrators	950
Jail administrators	950
Probation administrators	950
Community substance use treatment administrators	950

Note: The total survey population may be less than 3800 because some counties have the same person filling multiple roles listed above

To compile the respondent list, the research team developed a database of all Stepping Up and matched comparison counties. NACO-CSG-APAF provided a list of county contacts for Stepping Up counties. We contacted these individuals to provide the appropriate contact information for jail, probation, mental health, and substance use administrators in their county. We also conducted web-based searching. For comparison counties, we identified county-level experts through web-based searching. When contact information was not publicly available, we called individual agencies to identify the correct respondents. We also engaged in a snowball technique, in which we contacted experts already identified for assistance in identifying other possible respondents in their county.

#### Survey administration

The web-based survey is administered using Qualtrics. Using a procedure described by Dillman [34], respondents receive an introductory email that includes a NACO-CSG-APAF endorsement letter of support as well as key information to collect prior to beginning the survey (e.g., budget and staffing data). One week following administration of the introductory email, an invitation to participate in the survey is sent using Qualtrics. The research team sends follow-up emails once a week for 3 weeks following the initial invite with a reminder to participate. If the survey is not completed by the end of week 4, research team members make follow-up phone calls. During these calls, the research team provides multiple options for the respondents to complete the survey, including completion of the survey via telephone and receipt of a paper copy. Following the phone call, the research team continues to follow-up with respondents biweekly. Given the current context (i.e., COVID-19), we anticipate encouraging survey participation for 6 months before closing the survey. We will also provide county-specific feedback reports on county-level CJ behavioral health indicators as an incentive for study participation.

#### Survey validation

We use existing, validated measures where possible. When we needed to tailor items to CJ or to mental health, we used Cook [35] strategies for item development by testing new items using cognitive interviews [36]. Interviews covered question comprehension, decision processes, and response options.

Ten cognitive interviews were conducted in May 2020 with volunteers from Stepping up counties representing jail, probation/parole, community mental health, and community substance use treatment. Interviews were conducted via videoconference. Team members met to iteratively review interview results and revise the survey. At these meetings, each interviewer presented the responses and reflections from their interviews. Volunteer comments and interviewer/notetaker feedback, along with expert review by team members, were used to revise the survey. Changes were made to simplify and clarify survey questions and to remove redundancies. The amended survey was again reviewed by all team members.

### Measures

Measures will be collected at all 3 time points (see Table 3). All respondents will receive the same assessments. We refer to measures as “agency-level measures” when analyses of these measures will account for nesting within counties, but the primary focus is on agencies. We refer to measures as “agencies nested within counties” when we used nested analyses and our primary focus is on the county level. We refer to measures as “county-level” measures if they produce a single value for the county to be analyzed at the county level.

**Table 3:** Study assessments

Construct	Measures and indicators	Time point		
		Baseline	8 months	36 months
**Target implementation mechanisms**				
Use of/capacity for performance monitoring	Performance Monitoring measure (primary) Adapted Routine Decision-Making scale [37]	X X	X X	X X
Use and functioning of nteragency teams	Adapted NCJTP Relationship Assessment Inventory [38]	X	X	X
Common goals and mission across agencies	Adapted NCJTP Goals/Mission scale [38] (primary) Agreement (kappa) among respondents within counties on ratings of the importance of providing mental health treatment services for justice-involved individuals	X X	X X	X X
System integration (vs. single programs)	Use of same screening/assessment instruments across agencies in the county	X	X	X
	Total score of 18+ on the NCJTP Relationship Assessment Inventory [38]	X	X	X
**Implementation outcomes**				
Number of justice-involved clients who received behavioral health services (primary)	How many justice-involved individuals received any mental health service and how many received any substance use service in the agency in the past year	X	X	X
Number of behavioral health EBPP available	Questions asking whether each listed EBPP is available to justice-involved individuals in the county	X	X	X
Resources for behavioral health EBPP	Funding increases and decreases Staff numbers, roles, training NCJTPS’s Assess Your Resources scale [38]	X X X	X X X	X X X
**Covariates**				
Baseline values of dependent variables	As assessed above	X		
Months since county joined Stepping Up	Self-report and Stepping Up records	X		
Matching score	Matching score used to choose matched comparison counties	X		
Indicators of whether the county shares (a) their mental health administrator, and/or (b) justice administrators with other counties	Questions asking whether the county shares (a) their mental health administrator, and/or (b) justice administrators with other counties	X		
**Descriptors and moderators**				
Type of agency	NCJTP About Your Organization scale [38]	X	X	X
Staffing (type, number, urnover)	Adapted NCJTP Staffing scales [38]	X	X	X
Organizational support for innovation	Adapted NCJTP Assess Your Organizational Culture scale [38]	X	X	X
Policy context	State mental health diversion funding, legislative reforms	X	X	X

#### Descriptors, predictors, and moderators (agency level)

A series of measures will be used to describe the inner context of each agency. *Type of agency* will be characterized using the National Criminal Justice Treatment Practices (NCJTP) survey About Your Organization scale [38]. *Staffing*, including type, number, and turnover, will be measured using the adapted NCJTP Staffing scales [38]. *Organizational Culture Support for Innovations* (a proposed moderator) will be assessed using an adapted version of the NCJTP Assess Your Organizational Culture scale [38].

#### Aim 1: Target mechanisms

##### Use of and capacity for performance monitoring (agencies nested within counties)

We created a Performance Monitoring measure which provides one point for each of the following: (1) whether counties are *able* to measure the 4 Stepping Up core metrics (number of mentally ill people who are booked into jail, average length of jail stay, percent who are connected to community-based treatment upon release from jail, and rate of return to jail) (up to 4 points); (2) each metric they regularly report (up to 4 points); and (3) each metric used for ongoing decision making (up to 4 points) (up to 12 points total). A secondary measure to capture performance monitoring identifies 7 kinds of decisions (e.g., budget preparation, medicine supply) and asks whether they were guided by each of the 4 Stepping Up core metrics (0 = no, 1 = yes), for up to 28 points possible. This measure was adapted from the Routine Decision-Making scale of the Performance of Routine Information System Management (PRISM) Toolkit [37].

##### Use and functioning of interagency teams (agencies nested within counties)

To examine the activities and functioning of interagency teams, we integrated the NCJTP Relationship Assessment Inventory [38] with additional items based on the goals and priorities of Stepping Up. This integrated scale contains 18 items such as “we share general information about populations in need of treatment services” (0 = no, 1 = yes) with one point assigned for each collaborative activity across the other 3 agencies. The total score (up to 54) reflects joint activities among agencies.

##### Common goals and mission across agencies (agencies nested within counties)

The primary measure (an adapted NCJTP Goals/Mission scale) [38] assesses each respondent’s perception of the degree to which their agency goals and overall county goals align. Respondents are given a list of goals (e.g., public safety/protection, provide mental health services) and are asked to rank them according to (1) their agency’s priorities and (2) county priorities. A kappa score reflects the degree of consistency between the two lists. The secondary measure, a county-level measure, will be agreement (kappa) among respondents within counties on ratings of the importance of providing mental health treatment services for justice-involved individuals in jail and in the community (separately) on a scale of 1 (unimportant) to 10 (important).

##### System integration (agencies nested within counties)

 is a dichotomization of the NCJTP Relationship Assessment Inventory [38] total score (i.e., excluding the additional items). Counties with scores of 18 or more are considered to have achieved “system integration”. A secondary (county level) measure will reflect the degree to which each of 12 listed behavioral health screening and assessment instruments are used by and/or shared among multiple responding agencies within a county. For each of the 12 instruments listed, counties will receive a score (0 = no agencies use the same instrument, 1 = two agencies use the same instrument, 2 = three agencies use the same instrument, and 3 = all four agencies use the same instruments).

#### Aim 2: implementation outcomes

##### Number of justice-involved adult clients receiving behavioral health services (agencies nested within counties; primary)

After defining “justice-involved” and asking whether each of the EBPP described below is available in the county, we ask respondents how many justice-involved individuals received any mental health service and how many received any substance use service in their agencies in the past year.

##### Number of behavioral health EBPP available to justice-involved individuals (county level)

Mental health EBPPs were taken from treatment recommendations for justice-involved individuals [39–45] and from community standards for treatment of serious mental illness posttraumatic stress disorder, borderline personality disorder, suicide thoughts or behaviors, anxiety, insomnia, and pain [46–52]. Substance use EBPPs were taken from the US National Institute on Drug Abuse’s consensus list [53]. Using the EBPP list described above, we ask whether each EBPP is available to justice-involved individuals in the county. If any of the respondents answers “yes”, we count that EBPP as being available to justice-involved individuals in the county.

##### Resources for behavioral health EBPP for justice-involved individuals (agency level)

Respondents will be asked to report whether their agency has experienced an increase (+ 1), no change (0), or decrease (− 1) in funding from the prior year in 13 different areas (e.g., “screening and assessment”). We will cluster these 13 areas using factor analysis and then create total scores for each factor, which will serve as primary outcome/s. Initially, we planned to assess the total dollar amount of resources devoted to behavioral health services for justice-involved individuals, but found that most agencies could not report this number. Secondary measures relate to capacity and training: (1) the proportion of staff in clinical roles, (2) how many staff participated in behavioral health-related training in the past year, and (3) number of staff hired minus the number who left in the prior year. Lastly, we will use the NCJTPS’s Assess Your Resources scale [38], which uses Likert scale items, to measure respondent perceptions of the adequacy of resources available in their agency.

#### Aim 3: characterize implementation processes and critical incidents

##### Qualitative

We will use qualitative data to triangulate quantitative findings, enrich our understanding of how the target mechanisms work and lead to outcomes and critical incidents to EBPP implementation success or failure. Qualitative data will include interviews with 30 of the 475 county pairs (60 paired counties total). County pairs were randomly selected at Wave 1 (stratified by small, medium, and large county populations) and followed longitudinally at Waves 2 and 3. We anticipate 180 qualitative interviews (60 respondents at 3 time points). We will alternate CJ and behavioral health respondents to obtain multiple perspectives on the county’s progress. Respondents will be invited for interviews regardless of their survey status (i.e., completed, not yet completed, declined) for that wave.

*Fidelity to Stepping Up/Quantitative characterization of implementation strategies and sub-strategies used* to improve mental health or substance use services for justice-involved individuals and/or to reduce the number of people with mental illness in jail (agencies nested within counties). We will use a checklist with strategies and their descriptions constructed from the six main Stepping Up strategies as well as categories conceptualized by Powell [54] and the CJ-IIM [17]. Respondents will select whether anyone in the county is “planning to address this”, “some progress made”, or “significant progress made” using each strategy.

### Power analyses

The expected sample (475 paired counties, up to 4 respondents per county, resulting sample size of ~ 3800) and the response rate of 50% gives an expected sample of 1900 respondents. Given that anywhere from 0 to 4 respondents may complete the survey in any given county, with a 50% overall response rate, we anticipate that 712 counties will have at least one respondent who completes the survey. We used a conservative (higher than expected) intraclass correlation coefficient of 0.1 for addressing clustering of agencies within counties.

For county-level analysis, an effect size of 0.2, power of 0.8, confidence level of 95%, and statistical significance level of 0.05 were used to calculate the minimum sample size. Repeated measures analysis required a minimum total sample size of 304 counties. For logistic regression and other nonlinear predictive models, depending on the type and quantity of variables used in the model, the minimum total sample size required varied between 156 and 489 counties. With attrition, our calculations showed a power of 0.9 and more for most county-level analyses.

For agency-level analysis, a conservative agency-level effect size estimate (*d* = 0.1), a power of 0.8, confidence level of 95%, and significance level of 0.05 were used to calculate the minimum required sample size. Repeated measures analysis comparing respondents from Stepping Up and comparison counties resulted in a minimum sample size of 524 respondents. When comparing the response measures, measured at the agency level, over time, a minimum sample size of 1200 respondents is required. For predictive logistic regression and other nonlinear predictive models, the minimum sample size varied between 673 and 1100 respondents. Given the larger sample size of this study, our calculations showed a power higher than 0.9 for agency-level analyses.

### Analysis plan

#### General approaches

Primary tests will be 2-sided with α = 0.05. Analysis approaches accommodate nested and repeated measures data. We will examine predictive associations between Stepping Up membership, hypothesized target mechanisms, and implementation outcomes over time. We will use general linear models and generalized linear mixed models (GLMM) when the dependent variables are continuous and noncontinuous, respectively. GEE will be used instead when distributional assumptions are not met. For non-aggregated dependent variables reported at the agency level (i.e., hierarchical data), a random intercept growth hierarchical linear model (GHLM) will be fitted. All analyses will covary: (1) Wave 1 (baseline) values of dependent variables, (2) months since the county joined Stepping Up, (3) the matching score, (4) an indicator representing whether a the county shares their mental health administrator with other counties, and (5) a similar indicator for shared justice roles across counties.

#### Missing data

We will review survey completeness and recontact respondents to address quality issues and increase response rates. Logistic regression will be used to determine the type of missingness. Within waves, multiple imputation techniques will be applied. To address issues of missing data across different waves (i.e., over time), we will use generalized estimating equations (GEE) or weighted GEE, depending on the type of missing data.

#### Aim 1a: comparison of target mechanisms between Stepping Up and non-Stepping Up counties

##### Primary

We will test the hypothesis that Stepping Up counties will show faster rate of improvement in *the use of/ capacity for performance monitoring* (i.e., total scores on our Performance Monitoring measure) than comparison counties, using GLMM or GEE. Analyses will test for differences in slopes (rates of change). Separate secondary analysis will compare rates of change in the adapted Routine Decision-Making scale total score between Stepping Up and comparison counties.

##### Secondary

We will test the hypothesis that Stepping Up counties will show faster rate of improvement in the *use/functioning of interagency teams* (i.e., total scores on the integrated NCJTP Relationship Assessment Inventory-IOR measure) than control counties using the same statistical techniques. We will test the hypothesis that Stepping Up counties will show faster rate of improvement in *common goals and mission across agencies* (i.e., agreement between perceived agency and county priorities) than comparison counties using GLMM or GEE. We will conduct similar analyses of agreement among respondents within each county on the importance of mental health treatment for justice-involved individuals. We will test the hypothesis that Stepping Up counties will show faster rate of improvement in *system integration* (score of 6 or more on the Relationship Assessment Inventory score) using GEE. Separate secondary analysis will compare rates of change in the use of the same screening and assessment instruments by multiple agencies in Stepping Up and comparison counties.

#### Aim 1b: tests of mediation

##### Primary

We will test the hypothesis that *changes in use of performance measures* (i.e., scores on UPMDC and the adapted Routine Decision-Making scale) will mediate any differences found in *rates of change in primary measures of justice-involved clients receiving behavioral health services, number of EBPPs, and resources available*. These primary mediator analyses will use structural equation models, and path analyses.

##### Secondary

We will conduct a series of analyses examining changes in interagency teams, common goals and missions, and integrated systems of care using scores from respective measures identified above, as mediators of a number of justice-involved individuals receiving services, number of EBPPs available, and number of resources using appropriate baseline measures or months since joining Stepping Up as controls.

#### Aim 2: comparison of implementation outcomes between Stepping Up and non-Stepping Up counties

##### Primary

We will test the hypothesis that Stepping Up counties will show faster rate of improvement in the number of justice-involved clients receiving behavioral health services than will comparison counties, using GLMM and GEE. Analyses will test for differences in slopes (rates of change) between the two sets of counties.

##### Secondary

We will separately test the hypotheses that Stepping Up counties will show faster rate of improvement in the *number of behavioral health EBPPs available to* and *resources for behavioral health EBPP for justice-involved individuals* using GLMM, GEE, and GHLM.

We will examine moderators of the effects of Stepping Up participation on our primary outcome (justice-involved clients receiving behavioral health services) using structural equation models. A priori moderators include months between a county joining Stepping Up and study baseline, levels of implementation outcomes at study baseline, type of agency, organizational culture support for innovations (i.e., score on NCJTP Assess Your Organizational Culture scale [38]), jails with their own behavioral health staff, yes/no presence of legislative reforms, counties in states that have mental health diversion funding, and counties with divisions that provide cross-system trainings.

#### Aim 3: characterize implementation processes and critical incidents and assess counties’ fidelity to Stepping Up (quantitative)

We will examine the relationships between use of implementation strategies identified in the Implementation Strategy Checklist and faster rates of change in implementation outcomes, using GLMM and GEE while controlling for baseline measures and months since joining Stepping Up. We will use Bonferroni correction to control for multiple comparisons of implementation strategies (using the Checklist) for each of the three implementation outcomes.

##### Fidelity

We will compare Stepping Up and comparison counties on rates of use of Stepping Up strategies as a measure of fidelity to the National Stepping Up program, and we will compare counties on rates of use of other strategies to explore whether Stepping Up impacts related strategies.

#### Aim 3: characterize implementation processes and critical incidents (qualitative)

Qualitative data will be analyzed in line with study aims and key research questions using a two-stage analysis plan. In Stage 1, after each interview, interviewers will summarize key topics in framework matrix [55], which allows key topics to be reviewed quickly. In Stage 2, recordings will be transcribed by a professional transcription service and will be anonymized before coding. *Deductive codes* will be drawn from interview question topics using the CJ-IIM, the 6 Stepping Up main strategies, and critical incidents. *Inductive codes* capturing emergent themes will arise from team-level review of transcripts. Coding team members will independently code transcripts; 20% will be double coded and reviewed for fidelity. Codes will be entered into NVivo^166^, using thematic^171^ analyses; an audit trail will be maintained through code development and analysis. We will compare patterns found in the qualitative data to patterns found in our quantitative data; this side-by-side comparison of patterns can identify sign posts for additional exploration and analyses.

## Discussion

This study will accelerate knowledge on how to implement mental health and substance use EBPPs in the setting of serving justice-involved individuals by evaluating target mechanisms, implementation outcomes, and strategies used in Stepping Up and comparison counties across the USA. This large study of an ongoing natural experiment is an unprecedented opportunity to evaluate multi-system CJ implementation efforts on a national scale. It will elucidate effective policies and strategies for bringing evidence-based practices to large, vulnerable, and underserved populations. Study rigor is improved through use of the CDC matching methodology and covarying baseline values in analyses. Analyses examine differences between conditions in within-county rates of change over time, increasing rigor for this quasi-experimental design. The quasi-experimental design is especially strong and the sample large (950 counties) for a policy implementation research study, a still understudied area of implementation science.

## Supplementary Information

**Additional file 1.** NIH Notice of Award.

**Additional file 2.** IRB exemption.

**Additional file 3.** STROBE checklist.

## Data Availability

Not applicable

## Abbreviations

APAF: American psychiatric association foundation
CDC: Centers for disease control and prevention
CJ: Criminal justice
CJ-IIM: Criminal justice interagency implementation model
CSG: Council of state governments
EBPP: Evidence-based practice or policy
GEE: Generalized estimating equation
GHLM: Growth hierarchical linear model
GLMM: Generalized linear mixed models
IOR survey: Inter-Organization relationship survey
NACo: National association of counties
NCJTP survey: National criminal justice treatment practices survey
PRISM Toolkit: Performance of routine information system management toolkit
UPMDC: Use of performance measures and data checklist
