# Supplementary material for: Protocol for a quasi-experimental, 950 county study examining implementation outcomes and mechanisms of Stepping Up, a national policy effort to improve mental health and substance use services for justice-involved individuals
Source: Implement Sci. 2021 Mar 29;16:31. doi: 10.1186/s13012-021-01095-2 (PMC8006626; doi:10.1186/s13012-021-01095-2)
Supplement: Supplementary file 1 — Additional file 1. NIH Notice of Award. [file 13012_2021_1095_MOESM1_ESM.pdf]

RESEARCH
Department of Health and Human Services
National Institutes of Health

Federal Award Date: 09/24/2019

NATIONAL INSTITUTE OF MENTAL HEALTH

Grant Number: 1R01MH118680-01A1 REVISED
FAIN: R01MH118680

Principal Investigator(s):
JENNIFER E JOHNSON, PHD
Faye S Taxman (contact), PHD

Project Title: Evaluation of Stepping Up Efforts to Improve MH Services and Justice Utilization

Senator, Donna
Associate Director, Proposal & Award Mgmt
4400 University Drive, MSN 4C6
Fairfax, VA 220304422

Award e-mailed to: mlaskofs@gmu.edu

Period Of Performance:
Budget Period: 09/03/2019 – 07/31/2020
Project Period: 09/03/2019 – 07/31/2024

Dear Business Official:

The National Institutes of Health hereby revises this award (see "Award Calculation" in Section I and "Terms and Conditions" in Section III) to GEORGE MASON UNIVERSITY in support of the above referenced project. This award is pursuant to the authority of 42 USC 241 42 CFR 52 and is subject to the requirements of this statute and regulation and of other referenced, incorporated or attached terms and conditions.

Acceptance of this award including the "Terms and Conditions" is acknowledged by the grantee when funds are drawn down or otherwise obtained from the grant payment system.

Each publication, press release, or other document about research supported by an NIH award must include an acknowledgment of NIH award support and a disclaimer such as "Research reported in this publication was supported by the National Institute Of Mental Health of the National Institutes of Health under Award Number R01MH118680. The content is solely the responsibility of the authors and does not necessarily represent the official views of the National Institutes of Health." Prior to issuing a press release concerning the outcome of this research, please notify the NIH awarding IC in advance to allow for coordination.

Award recipients must promote objectivity in research by establishing standards that provide a reasonable expectation that the design, conduct and reporting of research funded under NIH awards will be free from bias resulting from an Investigator's Financial Conflict of Interest (FCOI), in accordance with the 2011 revised regulation at 42 CFR Part 50 Subpart F. The Institution shall submit all FCOI reports to the NIH through the eRA Commons FCOI Module. The regulation does not apply to Phase I Small Business Innovative Research (SBIR) and Small Business Technology Transfer (STTR) awards. Consult the NIH website http://grants.nih.gov/grants/policy/coi/ for a link to the regulation and additional important information.

If you have any questions about this award, please contact the individual(s) referenced in Section IV.

Sincerely yours,

Theresa A. Mercogliano
Grants Management Officer
NATIONAL INSTITUTE OF MENTAL HEALTH

Additional information follows

SECTION I – AWARD DATA – 1R01MH118680-01A1 REVISED

Award Calculation (U.S. Dollars)

**Salaries and Wages**	$208,864
**Fringe Benefits**	$64,341
**Personnel Costs (Subtotal)**	$273,205
**Consultant Services**	$8,000
**Materials & Supplies**	$31,100
**Travel**	$4,100
**Subawards/Consortium/Contractual Costs**	$282,796
**Federal Direct Costs**	$599,201
**Federal F&A Costs**	$94,758
**Approved Budget**	$693,959
**Total Amount of Federal Funds Obligated (Federal Share)**	$693,959
**TOTAL FEDERAL AWARD AMOUNT**	$693,959
**AMOUNT OF THIS ACTION (FEDERAL SHARE)**	$0

SUMMARY TOTALS FOR ALL YEARS		
**YR**	**THIS AWARD**	**CUMULATIVE TOTALS**
1	$693,959	$693,959
2	$672,963	$672,963
3	$633,366	$633,366
4	$627,168	$627,168
5	$617,754	$617,754

Recommended future year total cost support, subject to the availability of funds and satisfactory progress of the project

Fiscal Information:

CFDA Name: Mental Health Research Grants
CFDA Number: 93.242
EIN: 1540836354A1
Document Number: RMH118680A
PMS Account Type: P (Subaccount)
Fiscal Year: 2019

IC	CAN	2019	2020	2021	2022	2023
MH	8032183	$693,959	$672,963	$633,366	$627,168	$617,754

Recommended future year total cost support, subject to the availability of funds and satisfactory progress of the project

NIH Administrative Data:
PCC: 82-SEMS / **OC**: 414A / **Released**: MERCOGL 09/23/2019
Award Processed: 09/24/2019 12:07:48 AM

SECTION II – PAYMENT/HOTLINE INFORMATION – 1R01MH118680-01A1 REVISED

For payment and HHS Office of Inspector General Hotline information, see the NIH Home Page at http://grants.nih.gov/grants/policy/awardconditions.htm

SECTION III – TERMS AND CONDITIONS – 1R01MH118680-01A1 REVISED

This award is based on the application submitted to, and as approved by, NIH on the above-titled project and is subject to the terms and conditions incorporated either directly or by reference in the following:

a. The grant program legislation and program regulation cited in this Notice of Award.
b. Conditions on activities and expenditure of funds in other statutory requirements, such as those included in appropriations acts.

c. 45 CFR Part 75.
d. National Policy Requirements and all other requirements described in the NIH Grants Policy Statement, including addenda in effect as of the beginning date of the budget period.
e. Federal Award Performance Goals: As required by the periodic report in the RPPR or in the final progress report when applicable.
f. This award notice, INCLUDING THE TERMS AND CONDITIONS CITED BELOW.

(See NIH Home Page at http://grants.nih.gov/grants/policy/awardconditions.htm for certain references cited above.)

Research and Development (R&D): All awards issued by the National Institutes of Health (NIH) meet the definition of "Research and Development" at 45 CFR Part§ 75.2**.** As such, auditees should identify NIH awards as part of the R&D cluster on the Schedule of Expenditures of Federal Awards (SEFA). The auditor should test NIH awards for compliance as instructed in Part V, Clusters of Programs. NIH recognizes that some awards may have another classification for purposes of indirect costs. The auditor is not required to report the disconnect (i.e., the award is classified as R&D for Federal Audit Requirement purposes but non-research for indirect cost rate purposes), unless the auditee is charging indirect costs at a rate other than the rate(s) specified in the award document(s).

This institution is a signatory to the Federal Demonstration Partnership (FDP) Phase VI Agreement which requires active institutional participation in new or ongoing FDP demonstrations and pilots.

An unobligated balance may be carried over into the next budget period without Grants Management Officer prior approval.

This grant is subject to Streamlined Noncompeting Award Procedures (SNAP).

This award is subject to the requirements of 2 CFR Part 25 for institutions to receive a Dun & Bradstreet Universal Numbering System (DUNS) number and maintain an active registration in the System for Award Management (SAM). Should a consortium/subaward be issued under this award, a DUNS requirement must be included. See http://grants.nih.gov/grants/policy/awardconditions.htm for the full NIH award term implementing this requirement and other additional information.

This award has been assigned the Federal Award Identification Number (FAIN) R01MH118680. Recipients must document the assigned FAIN on each consortium/subaward issued under this award.

Based on the project period start date of this project, this award is likely subject to the Transparency Act subaward and executive compensation reporting requirement of 2 CFR Part 170. There are conditions that may exclude this award; see http://grants.nih.gov/grants/policy/awardconditions.htm for additional award applicability information.

In accordance with P.L. 110-161, compliance with the NIH Public Access Policy is now mandatory. For more information, see NOT-OD-08-033 and the Public Access website: http://publicaccess.nih.gov/.

In accordance with the regulatory requirements provided at 45 CFR 75.113 and Appendix XII to 45 CFR Part 75, recipients that have currently active Federal grants, cooperative agreements, and procurement contracts with cumulative total value greater than $10,000,000 must report and maintain information in the System for Award Management (SAM) about civil, criminal, and administrative proceedings in connection with the award or performance of a Federal award that reached final disposition within the most recent five-year period. The recipient must also make semiannual disclosures regarding such proceedings. Proceedings information will be made

publicly available in the designated integrity and performance system (currently the Federal Awardee Performance and Integrity Information System (FAPIIS)). Full reporting requirements and procedures are found in Appendix XII to 45 CFR Part 75. This term does not apply to NIH fellowships.

Treatment of Program Income:
Additional Costs

SECTION IV – MH Special Terms and Conditions – 1R01MH118680-01A1 REVISED

Clinical Trial Indicator: No
This award does not support any NIH-defined Clinical Trials. See the NIH Grants Policy Statement Section 1.2 for NIH definition of Clinical Trial.

FUNDING REVISION
This grant is revised for the sole purpose of providing a fully-funded commitment for FY2020.

THIS REVISED AWARD SUPERSEDES THE NOTICE OF AWARD ISSUED ON 9/3/19**.** THE FOLLOWING TERMS & CONDITIONS REMAIN IN EFFECT.

AWARD NOTICE:
This award has been made in response to the application submitted under the Funding Opportunity Announcement par-18-007 which can be referenced at:https://grants.nih.gov/grants/guide/pa-files/PAR-18-007.html

ADMINISTRATIVE REDUCTION:
In order to meet Institute program objectives within Fiscal Year 2019 budget constraints, future year recommended levels of support for this grant have been reduced by 5%.

CONSORTIUM / CONTRACTUAL COSTS:
This award includes funds for consortium activity with Michigan State University and Miriam Hospital. Each consortium is to be established and administered in accordance with the NIH Grants Policy Statement (http://grants.nih.gov/grants/policy/nihgps/index.htm). No foreign performance site may be added to this project without the written prior approval of the National Institute of Mental Health.

PARTICIPANT RECRUITMENT - MILESTONES:
Future NIMH support for this study is contingent upon adequate participant recruitment based on projected milestones as approved in the Recruitment Milestone Reporting system (RMR) on 8/22/19. It is expected that 3640 of the 10,380 total projected participants will be recruited by 4/1/20. This tri-yearly recruitment report should be submitted electronically to NIMH after each milestone period of April 1, August 1 and December 1 at: http://wwwapps.nimh.nih.gov/rmr/displayHome.action. ; In the event that actual recruitment falls significantly below projected milestones, NIMH may consider withholding future support and/or negotiating an orderly phase-out of this study. Information regarding the NIMH Policy for the Recruitment of Participants in Clinical Research is available at: http://grants.nih.gov/grants/guide/notice-files/NOT-MH-05-013.html.

BUDGET/PROJECT PERIOD ADJUSTMENT:
This grant has been selected under the NIMH plan to redistribute grant workloads more evenly throughout the year. Consequently, the initial budget period reflects a 7/31/20 end date. Subsequent budget periods will begin on August 1st and will be for a 12-month duration. Although this grant will have a slightly shorter budget period this year, it is awarded the full 12-month level of funds for the budget period. If needed, additional time may be requested at the end of the project period for a first no-cost extension through eRA Commons.

DATA AND SAFETY MONITORING:
The level and frequency of human subject data and safety monitoring should always be commensurate with the risk and nature of the research. Human subject data and safety monitoring for this grant is the responsibility of the Principal Investigator(s) and the IRB of record for this grant. The recipient is reminded of required reporting pursuant to NIHGPS 4.1.15.3 and the NIMH Reportable Events Policy cited in NOT-MH-19-027, as appropriate.

STAFF CONTACTS

The Grants Management Specialist is responsible for the negotiation, award and administration of this project and for interpretation of Grants Administration policies and provisions. The Program Official is responsible for the scientific, programmatic and technical aspects of this project. These individuals work together in overall project administration. Prior approval requests (signed by an Authorized Organizational Representative) should be submitted in writing to the Grants Management Specialist. Requests may be made via e-mail.

Grants Management Specialist: Theresa A. Mercogliano
Email: theresa.mercogliano@nih.gov **Phone**: 301-451-4940 **Fax**: 301-480-1956

Program Official: Denise M. Juliano-bult
Email: djuliano@mail.nih.gov **Phone**: 301-443-1638 **Fax**: 301-443-4045

SPREADSHEET SUMMARY
GRANT NUMBER: 1R01MH118680-01A1 REVISED

INSTITUTION: GEORGE MASON UNIVERSITY

Budget	Year 1	Year 2	Year 3	Year 4	Year 5
Salaries and Wages	$208,864	$230,801	$219,261	$219,261	$218,375
Fringe Benefits	$64,341	$60,548	$57,521	$57,521	$68,103
Personnel Costs (Subtotal)	$273,205	$291,349	$276,782	$276,782	$286,478
Consultant Services	$8,000	$8,000	$7,600	$7,600	$7,600
Materials & Supplies	$31,100	$17,800	$12,350	$7,600	$950
Travel	$4,100	$8,200	$7,790	$7,790	$7,790
Subawards/Consortium/Contractual Costs	$282,796	$262,516	$249,668	$249,455	$236,203
TOTAL FEDERAL DC	$599,201	$587,865	$554,190	$549,227	$539,021
TOTAL FEDERAL F&A	$94,758	$85,098	$79,176	$77,941	$78,733
TOTAL COST	$693,959	$672,963	$633,366	$627,168	$617,754

Facilities and Administrative Costs	Year 1	Year 2	Year 3	Year 4	Year 5
F&A Cost Rate 1	26%	26%	26%	26%	26%
F&A Cost Base 1	$364,454	$327,300	$304,522	$299,772	$302,818
F&A Costs 1	$94,758	$85,098	$79,176	$77,941	$78,733
