# Supplementary material for: Protocol for a quasi-experimental, 950 county study examining implementation outcomes and mechanisms of Stepping Up, a national policy effort to improve mental health and substance use services for justice-involved individuals
Source: Implement Sci. 2021 Mar 29;16:31. doi: 10.1186/s13012-021-01095-2 (PMC8006626; doi:10.1186/s13012-021-01095-2)
Supplement: Supplementary file 2 — Additional file 2. IRB exemption. [file 13012_2021_1095_MOESM2_ESM.pdf]

Office of Research Integrity and Assurance

Research Hall, 4400 University Drive, MS 6D5, Fairfax, Virginia 22030
Phone: 703-993-5445; Fax: 703-993-9590

DATE:	October 7, 2020
TO:	Faye Taxman, PhD
FROM:	George Mason University IRB
Project Title:	[1615083-4] Evaluation of Stepping Up: Comparing Stepping Up Counties to a Matched Sample
Reference:	OSP #204928
SUBMISSION TYPE:	Amendment/Modification
ACTION:	DETERMINATION OF EXEMPT STATUS
DECISION DATE:	October 7, 2020
REVIEW CATEGORY:	Exemption category #2

Thank you for your submission of Amendment/Modification materials for this project. The Institutional Review Board (IRB) Office has determined this project is EXEMPT FROM IRB REVIEW according to federal regulations.

You are required to follow the George Mason University Covid-19 research continuity of operations guidance. You may not begin or resume any face-to-face interactions with human subjects until (i) Mason has generally authorized the types of activities you will conduct, or (ii) you have received advance written authorization to do so from Mason's Research Review Committee. In all cases, all safeguards for face-to-face contact that are required by Mason's COVID policies and procedures must be followed.

Please remember that all research must be conducted as described in the submitted materials.

Please note that any revision to previously approved materials must be submitted to the IRB office prior to initiation. Please use the appropriate revision forms for this procedure.

If you have any questions, please contact Bess Dieffenbach at 703-993-5593 or edieffen@gmu.edu. Please include your project title and reference number in all correspondence with this committee.

Please note that all research records must be retained for a minimum of five years, or as described in your submission, after the completion of the project.

Please note that department or other approvals may also be required to conduct your research.

GMU IRB Standard Operating Procedures can be found here: https://oria.gmu.edu/topics-of-interest/human-subjects/

Generated on IRBNet

This letter has been electronically signed in accordance with all applicable regulations, and a copy is retained within George Mason University IRB's records.

Generated on IRBNet
